# A Novel Derivative of (-)mycousnine Produced by the Endophytic Fungus *Mycosphaerella nawae*, Exhibits High and Selective Immunosuppressive Activity on T Cells

**DOI:** 10.3389/fmicb.2017.01251

**Published:** 2017-07-05

**Authors:** Li-Wei Wang, Jin-Liang Wang, Jing Chen, Jia-Jie Chen, Jia-Wei Shen, Xiao-Xiao Feng, Christian P. Kubicek, Fu-Cheng Lin, Chu-Long Zhang, Feng-Yang Chen

**Affiliations:** ^1^Department of Pharmaceutical Science, College of Medical Science, Hangzhou Normal UniversityHangzhou, China; ^2^State Key Laboratory of Rice Biology, Institute of Biotechnology, Zhejiang UniversityHangzhou, China; ^3^Institute of Chemical Engineering, Vienna University of TechnologyVienna, Austria; ^4^Institute of Materia Medica, Zhejiang Academy of Medical SciencesHangzhou, China; ^5^Department of Basic Medical Science, Hangzhou Medical CollegeHangzhou, China

**Keywords:** *Mycosphaerella nawae*, (-)mycousnine enamine, dibenzofurane, T-cell targeting, immunosuppressive activity

## Abstract

An endophytic fungus, *Mycosphaerella nawae* ZJLQ129, was isolated from the leaves of the traditional Chinese medicine Smilax china. From the fermentation broth and mycelium, a dibenzofurane compound (-)mycousnine (1) was isolated. Chemical modification of it to the amide derivative (-)mycousnine enamine (2), which is new to science, was found to have high and selective immunosuppressive activity: similar to cyclosporin A, (-)mycousnine enamine (2) selectively inhibited T cell proliferation, suppressed the expression of the surface activation antigens CD25 and CD69 and the formation and expression of the cytokines interleukin-2 as well as interferon γ in activated T cells, but did not show any effect on the proliferation of B cells and cancer cells (PANC-1 and A549) and the activation of macrophages. Furthermore, the cytotoxicity of (-)mycousnine enamine was lower than that of cyclosporin A, and its therapeutic index (TC50/EC50) was 4,463.5, which is five-fold higher than that of cyclosporin A. We conclude that (-)mycousnine enamine (2), the semi-synthestic product prepared from the native product (-)mycousnine (1) of the endophyte M. nawae is a novel effective immunosuppressant showing low toxicity and high selectivity.

## Introduction

T lymphocytes are a critical component of the adaptive immune system, which play a key role in mediating various kinds of immune responses, thus T cell-directed therapies serve as logical and major treatment for autoimmune and inflammatory disorders, including transplant rejection, systemic lupus erythematosus, rheumatic arthritis and multiple sclerosis (Dimeloe et al., 2016). Various treatment modalities such as blockage of T cell receptor (TCR) signaling pathways, intervention of costimulatory and accessory molecules, T cell depletion, alteration of T cell adhesion and trafficking as well as modulation of cytokines have been designed and clinically applied for treatment of autoimmune and inflammatory diseases with varying degrees of success (Steward-Tharp et al., 2010). However, undesirable toxic side effects are still major obstacles in the use of these immunosuppressive agents (Smith et al., 2003; Clay and Pandya, 2011).

These severe side effects of most immunosuppressive agents are primarily due to their poor selectivity (Kahan, 2003; Halloran, 2004). As a consequence, there continues to be a high demand for new effective immunosuppressants which specifically inhibit T cell response without adverse reaction on other cell types and organs. Natural products and natural-product-like compounds provide a large reservoir of pharmaceutical material for the development of new drugs (Newman and Cragg, 2012; Mehbub et al., 2016). As a consequence, the mining of novel T-cell targeting immunosuppressants from the pool of naturally occurring micro-molecules is of great interest. In previous studies, we have isolated dozens of natural products from plants that exhibited immunosuppressive activities both *in vitro* and *in vivo* (Ye et al., 2006, 2008; Chen et al., 2010, 2012, 2015). However, most of these natural products lacked the desired selective properties.

Endophytic fungi are microbes living inside the internal tissue of the healthy plants without causing any obvious symptoms. They are potential source of novel medically important natural products with diverse antimicrobial, anti-inflammatory, antiviral, antitumor, anti-malarial or immunosuppressive activities (Strobel et al., 2004; Nisa et al., 2015; Liu et al., 2016). Endophytic fungi are thus considered a promising reservoir of natural products with interesting medical bioactivities. In support of this, we have recently identified several natural products with antimicrobial and antitumor activities from endophytic fungi (Wang et al., 2012, 2014).

*Smilax china* L., a member of the *Liliaceae* family, is a popular traditional Chinese medicine with the anti-inflammatory (Shao et al., 2007; Inamullah et al., 2009), antioxidative (Soom, 2011; Zhang and Guo, 2012), antitumor (Xu et al., 2008; Wu and Wang, 2010) and glucose-lowering activity for diabetic patients (Min et al., 2016) activities. In China, it has been extensively used for clinical treatment of furunculosis, selected tumors and inflammation (Xu et al., 2008; Song et al., 2017). However, to date and our knowledge, there are no reports regarding secondary metabolites of its endophytic fungi.

Dibenzofurane metabolites are an interesting kind of compounds originally isolated from lichens and endophytic fungi which have been shown to have antibiotic, antimycobacterial, antiviral, analgesic, or antipyretic properties (Mouafo et al., 2014; Marion et al., 2016; Yu et al., 2016). Thus, there is considerable interest in dibenzofuranes as potential sources of pharmacological agents.

In the course of our ongoing effort to investigate valuable compounds from fungal endophyte, we isolated a strain of *Mycospaerella nawae* (ZJLQ129) from healthy leaves of *Smilax china* L., which produces dibenzofuran compound (-)mycousnine. We converted it to its so far unknown amide derivative (-)mycousnine enamine, which has higher stability and exhibited enhanced,selective inhibition of the T cell response than (-) mycousnine. This component is thus a new candidate for developing immunosuppressive agent.

## Materials and methodsds

### Isolation and culture of endophytic fungi

Healthy leaves of the medicinal plant *S. china* were collected from LongQuan County of Zhejiang Province, China. The leaves were collected, placed in a plastic bag and then refrigerated for transportation to the lab within 24 h. Samples were leached with tap water to remove soil. The surface of the sample leaf was sterilized in 75% ethanol for one min, then followed by 0.5% (w/v) NaClO for ten min, and then rinsed four times in aseptic distilled water. After that it was cut into 10 × 10 mm pieces, and the pieces were placed on water agar (WA) (containing 100 μg/mL of kanamycin and ampicillin respectively) and then incubated at room temperature for a period between 3 and 15 days. The hypha tip was removed and placed on potato dextrose agar (PDA), incubated at room temperature and transferred to sub-culture on PDA as soon as they outcrop off the medium until pure colonies were formed.

### Fungal identification

Genomic DNA of the fungal strain ZJLQ129 was extracted by the CTAB method (Zhang et al., 2010). Internal transcribed spacer regions 1 and 2 (ITS1 and ITS2) of the rDNA was amplified by primers ITS1 (5′-TCCTCCGCTTATTGATATGC-3′) and ITS4 (5′-TCCTCCGCTTATTGATATGC-3′) (White et al., 1990) and sequenced in an ABI 3730 sequencer with the same primers. The sequence was deposited in GenBank (accession no.KX881942) and analyzed by BLAST. Sequences from species with highest similarity were retrieved. Sequence alignment was performed with Clustal x 1.81 and phylogenetic analyses conducted by Bayesian inference (BI) analyses using MrBayes v.3.2.6 (Luo et al., 2015). *Asteroma alneum* was chosen as the outgroup taxon. The fungal strain ZJLQ129 was deposited in the China Center for Type Culture Collection (CCTCC) as CCTCC M2015517.

### Fermentation and compound isolation

Strain ZJLQ129 was grown in 1 L Erlenmeyer flasks containing 500 mL potato dextrose broth (PDB, 40 L in total) medium. The flasks were cultured without shaking for 14 days at room temperature. Fungal cells and broth were separated by filtration, and the filtrate extracted three times with an equal volume of ethyl acetate (EtOAc). The extract was then vacuum-rotary evaporated to dryness to obtain Part A (total amount 1.72 g). The mycelium was thoroughly crushed in a mortar and then extracted three times with 80% acetone-H_2_O (v/v). The solution was condensed under reduced pressure an aqueous solution, which was then extracted 3 times with an equal volume of EtOAc to give part B (total amount 3.50 g). Parts A and B were combined to obtain the crude extract of (5.22 g). For isolation of potential bioactive compounds, the total crude extract was mixed with 16 g 100 mesh silica gel, dried at 50°C, and then loaded on a column of silica gel (200–300 mesh, 80 b 800 mm) The column was eluted with a linear gradient (8 L) of petroleum ether: ethyl acetate (75:25 to 0:100 v/v). Ninety fractions of 100 mL were collected and concentrated, then analyzed by thin-layer chromatography (TLC) on 25.4 × 76.2 mm silica gel G254 to combine the fractions with same components into eight fractions (F1-F8), The fraction F8 containing main metabolites (1.71 g) was then fractionated by Sephadex LH-20 column chromatography (CC) using MeOH/CHCl_3_ (1:1, v/v, 1 L) as a mobile phase. Twenty fractions of 5 mL were collected. Fractions F10–F14 were combined and recrystallized to yield a 511 mg of (-) mycousnine (for identification see below), which was unstable and started to undergo modification within 48 h at room temperature (monitored by TLC. First, a small drop of a solution of the pure compound is placed on three thin layer chromatography plates respectively. The first plate was run as soon as the compound spot had been placed on it using petroleum ether: chloroform: methanol (5:5:1 v/v/v) as mobile phase. The second and the third plates were trun 23 and 47 h later respectively in the same condition. Then the plates were placed in an enclosed container along with a fewiodine crystals. As a result, one, two and more than two brownish spots showed up on the first to the third plate, respectively. This result indicated the modification of mycousnine within 48 h). Working with it therefore started immediately after isolation into −20°C refrigerator.

### Synthesizing a stable derivate of (-)mycousnine

(-)mycousnine is a dibenzofurane metabolite with a highly reactive β-diketone system, which causes the observed instability (Kutney and Sanchez, 1976). In order to stabilize the compound, we decided to convert the -COCH_3_ substituent at position 2 in (-)mycousnine into an enamine group. This was done as follows: (-)mycousnine (100 mg) were dissolved in 8 mL methanol and then 2 mL concentrated ammonium hydroxide (25%, v/v) was added and the mixture was refluxed in a sealed tube for 12 h. After completion of the reaction (monitored by TLC), the solvent was removed under vacuum and the reaction product isolated by silica gel CC using petroleum ether: chloroform: methanol (180:40:10 v/v/v) as eluent to yield (-)mycousnine enamine (62 mg).

### Chemical and physical analyses

^13^C, ^1^H, and two-dimensional nuclear magnetic resonance (2D-NMR) as well as distortionless enhancement by polarization transfer (DEPT) spectrum were recorded on a BrukerDRX500 spectrometer using TMS as an internal standard. Coupling constants were recorded as *J* in Hertz and chemical shifts as δ values (parts per million). Melting points (MP) were detected on a Beijing X4 micro-melting point instrument. High-resolution mass spectra (HRESIMS) were tested on an Apex III Fourier-transforming ion cyclotron resonance (FTICR) Bruker mass spectrometer. Optical rotations ([α]20 D) were tested using a JASCO DIP-360 digital polarimeter. Single-crystal X-ray diffraction data were measured using a Bruker APEX2 diffractometer with graphite-monochromated Cu K radiation = 1.54178 Å. The crystallographic data for (-)mycousnine enamine in this paper have been deposited in the Cambridge Crystallographic Data Centre (CCDC) as supplementary publication number CCDC 1512383.

### Reagents

3-[4,5-dimethylthylthiazol-2-yl]-2,5-diphenyltetrazolium bromide (MTT), Concanavalin A (Con A), Cyclosporin A (CsA), dexamethasone (Dex), lipopolysaccharide (LPS), penicillin, and streptomycin were obtained from Sigma Chemical Co. in USA; Fetal bovine serum (FBS) was obtained from Hangzhou Sijiqing Corp. in China. Dulbecco's modified Eagle's medium (DMEM) was purchased from Thermo Fisher Scientific Inc. in USA. FITC-anti-CD3, PE-anti-CD25 and PE-anti-CD69 were obtained from eBioscience Inc. in USA; mouse cytokine (IL-2 and IFN-γ) detecting ELISA kits were purchased from Wuhan Boster Biological Technology., Ltd. In China; Trizol was obtained from Invitrogen in USA; PCR primers and reagents were obtained from Shanghai Sangon Biological Engineering Technology & Services Co., Ltd. in China.

### Experimental animals

Six weeks old female C57BL/6 mice were obtained from Shanghai Slac Laboratory Animal Co. Ltd. in China and acclimatized for 7 days before use. Mice were housed in the animal care facilities of Zhejiang Academy of Medical Sciences under pathogen-free conditions. Rodent laboratory chow and tap water were provided *ad libitum*, and maintained under controlled conditions: temperature 24 ± 1°C, humidity 50 ± 10%, 12-h light/12-h dark cycle. All the procedures were in strict accordance with The People's Republic of China legislation on the use and care of experimental animals, as well as the guidelines established by the Experimental Animals Center of Zhejiang Province. They were also approved by the Animal Care and Use Committee of Zhejiang Academy of Medical Sciences in China.

### Splenocytes proliferation assay

Splenocytes were prepared from female C57BL/6 mice, and Con A (3 μg/mL) or LPS (3 μg/mL) induced splenocytes proliferation was detected by MTT assay as previously described (Chen et al., 2012). The proliferation index was calculated by dividing the absorbance value for mitogen-cultures by the absorbance value for non-stimulated cultures.

### Cytotoxicity analysis

Splenocytes (5 × 10^6^ cell/mL) were seeded into four wells of a 96-well flat-bottom plate in 100 μl complete medium (DMEM medium supplemented with 10% heat-inactivated FBS, 100 U/ml penicillin and 100 μg/mL streptomycin), thereafter (-)mycousnine enamine was added giving a final volume of 200 μL. The plates were incubated at 37°C in a humidity atmosphere with 5% CO_2_. Cells were cultured for 48 h, and then cell viability was detected by MTT assay.

### Cell line and cell culture

Mouse macrophage RAW264.7, Human pancreas carcinoma cell line PANC-1 and lung adenocarcinoma cell line A549 were obtained from the Shanghai Institute for Biological Sciences, Chinese Academy of Sciences, and then cultured in complete medium. All cells were cultured in humidity air containing 5% CO_2_ at 37°C.

### Cell growth assay

PANC-1 or A549 cells (8 × 10^3^), which were harvested during the period of logarithmic growth, were seeded into each well of a 96-well flat-bottom plate and incubated overnight. The cells were treated with for 48 h with (-)mycousnine enamine thereafter and the cell viability were measured by MTT assay.

### LPS induced TNF-α production of Raw264.7 cells

Raw264.7 cells were seeded into 24-well flat-bottom plate at 1 × 10^5^ cell/mL in 1 ml complete medium, thereafter LPS (5 ng/mL) and (-)mycousnine enamine were added giving a final volume of 2 ml. After incubation for 24 h, the cultured supernatants were collected for the quantification of TNF-αusing a commercial ELISA kit (Sun et al., 2015).

### Analysis of CD3+ T cells proliferation

Splenocytes were seeded into 24-well flat-bottom plate at 5 × 106 cell/mL in 1 mL complete medium, thereafter Con A (3 μg/mL) and (-)mycousnine enamide were added to give a final volume of 2 mL. The plates were incubated for 48 h. The cells were collected and stained with FITC-anti-CD3. After washed with PBS, samples were immediately analyzed by a FACScan flow cytometer (Chen et al., 2015).

### Analysis of CD69 and CD25 cell surface markers

Splenocytes were seeded into 24-well flat-bottom plate at 5 × 106 cell/mL in 1 mL complete medium, thereafter Con A (3 μg/mL) and (-)mycousnine enamide were added giving a final volume of 2 mL. After incubation for 6 and 24 h, the cells were collected and stained with FITC-anti-CD3 plus PE-anti-CD69 and FITC-anti-CD3 plus PE-anti-CD25, respectively. After washing with PBS, the samples were immediately analyzed in a FACScan flow cytometer, respectively (Chen et al., 2015).

### Measurement of cytokines production and mRNA expression

Splenocytes were seeded into 24-well flat-bottom plate at 5 × 106 cell/mL in 1 mL complete medium, thereafter Con A (3 μg/mL) and (-)mycousnine enamide were added to give a final volume of 2 mL. After incubation for designated length of time, the cells and their supernatants were collected, and used for the detection of cytokines contents and mRNA expressions, respectively. The concentrations of IL-2 and IFN-γ in the culture supernatants were assayed by commercial ELISA kits. The collected cells were lysed with Trizol reagent and the total RNA isolated according to the manufacturer's protocol. The total RNA was reverse-transcribed into cDNA by using oligo(dT) primers. Real-Time PCR was performed using the SYBR Green PCR master mix. The primer pairs used in real-time PCR were the following: IFN-γ, 5′-CTGCTGATGGGAGGAGATG-3′, 5′-TTTGTCATTCGGGTGTAGTCA-3′; IL-2, 5′-TCAGCAACTGTGGTGGACTT-3′, 5′-GCCTTATGTGTTGTAAGCAGGA-3′; GAPDH, 5′-GGTTGTCTCCTGCGACTTCA-3′, 5′-TGGTCCAGGGTTTCTTACTCC-3′; GAPDH was used as an endogenous control. Primer amplification efficiency and specificity were verified for each set of primers. The mRNA expression levels of the tested genes relative to GAPDH were determined using the 2ΔΔCt method (Chen et al., 2015) and as fold induction.

### Statistical analysis

The data were expressed as mean ± standard deviation (SD). Statistical analyses were performed using ANOVA as well as the standard's *t*-test with SPSS data analysis software (version 13.0). *P*-values of less than 0.05 were regard as statistically significant.

## Results

### Identification of the endophytic fungus

**Figure 1:**
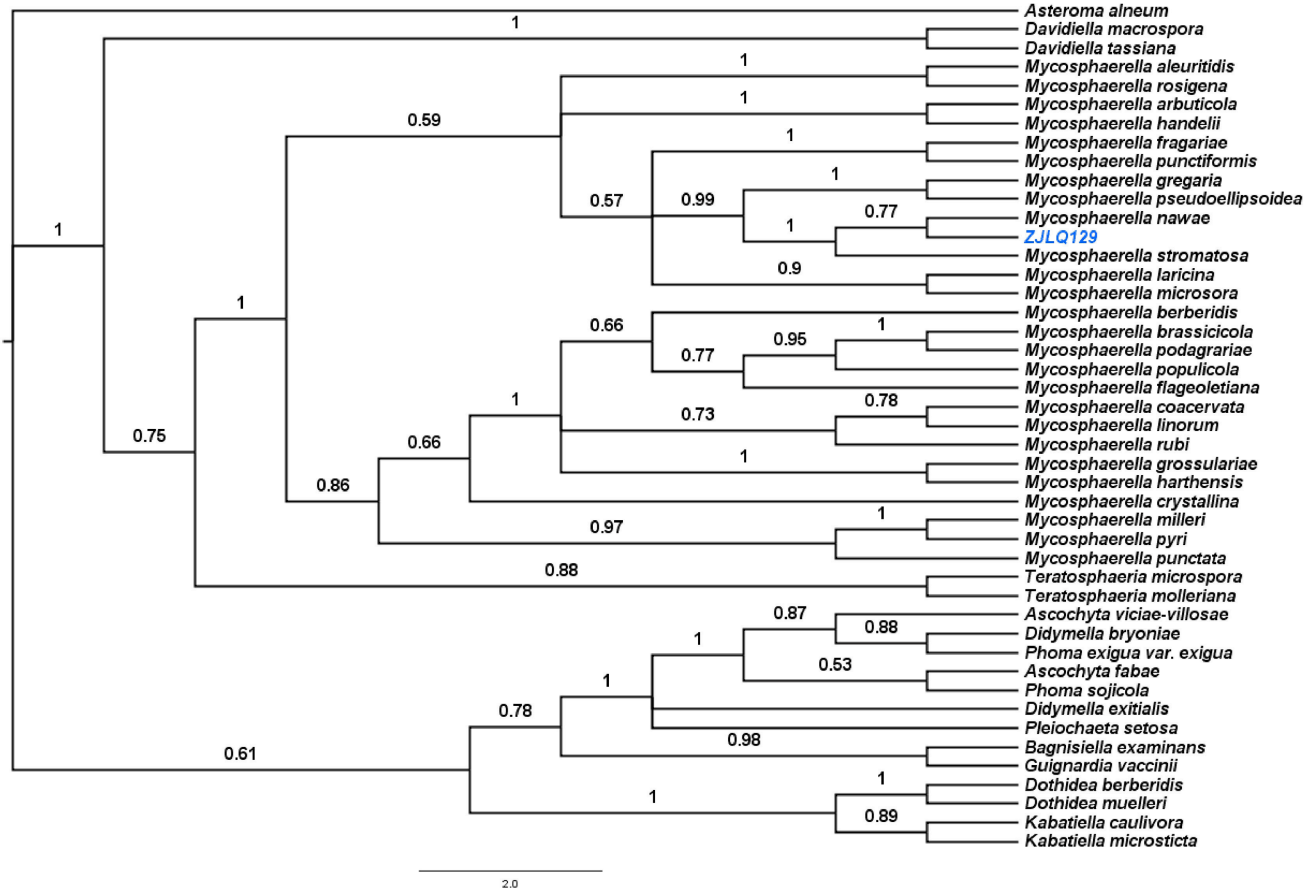
Bayesian phylogenetic tree based on the ITS1–5.8S–ITS2 sequences of the isolate ZJLQ129 and closest matches. *Asteroma alneum* is defined as an out-group. Bootstrap values are given for branches of the tree supporting the clades.

In order to identify the endophytic fungus that we isolated from the leaves of *Smilax china* L., ZJLQ129, we amplified its ITS1-5.8SrRNA-ITS2 genomic region. BLAST analysis of the corresponding sequence revealed several *Mycosphaerella* spp. as next neighbors. In order to identify the isolate at the species level, we aligned its ITS1-5.8rRNA-ITS2 with that of several species of *Mycosphaerella* and the phylogenetically close genera *Teratosphaeria, Ascochyta, Didymella, Phoma, Guignardia, Dothidea*, and *Kabatiella* (Simon et al., 2010). Analysis by Bayesian inference placed ZJLQ129 within a clade including all species of *Mycosphaerella*. Posterior probability values of the clade containing isolate ZJLQ129, *M. nawae* and *M. stromatisa* and of the subclade containg isolate ZJLQ129 and *M. nawae* is 1 and 0.77, respectively in the consensus tree indicate that ZJLQ129 is co-specific with *M. nawae* (Figure 1).

### Identification of the major metabolite produced by *M. nawae* and its stabilized product

**Figure 2:**
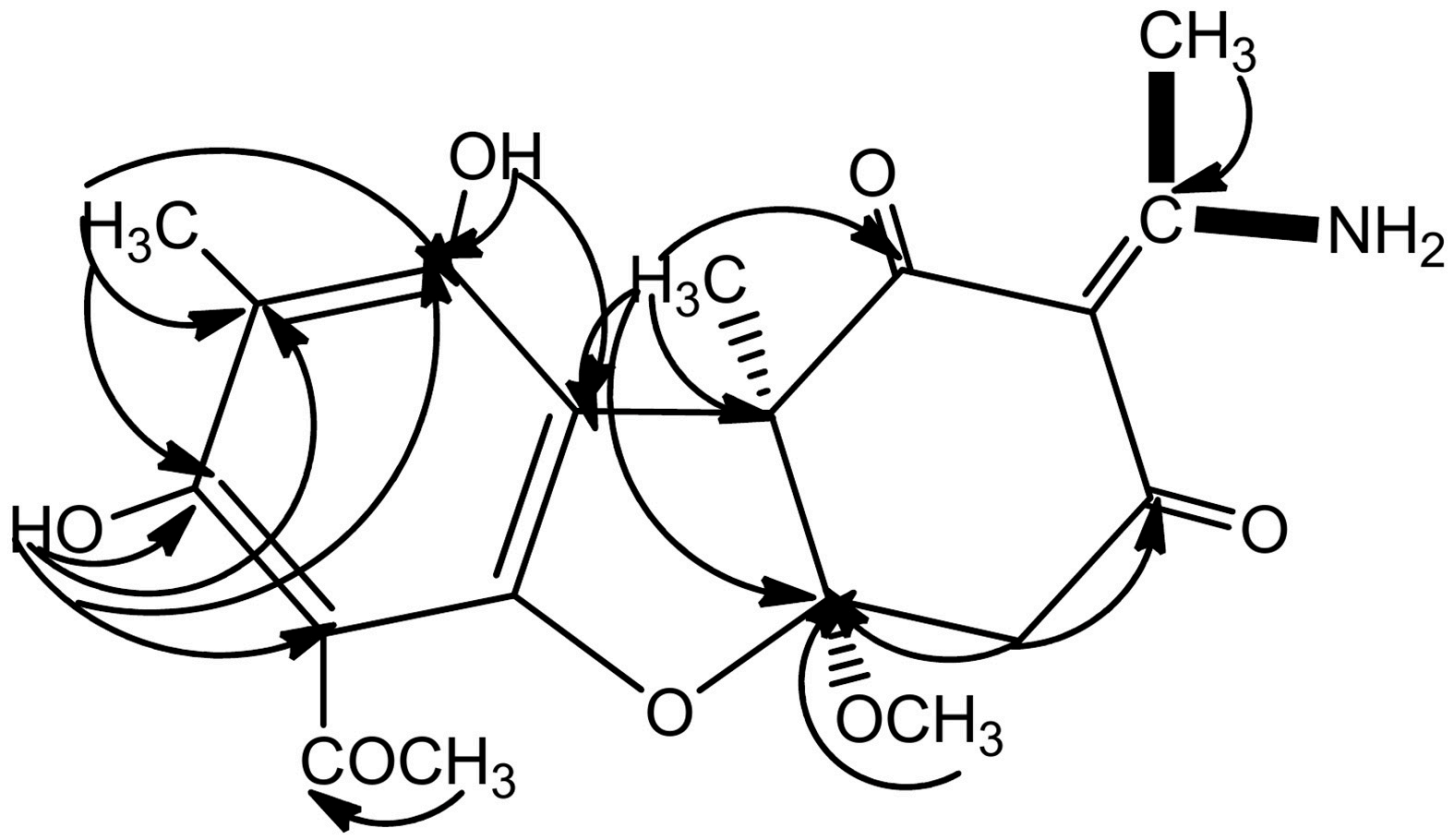
Key ^1^H-^1^H COSY and HMBC correlation of (-)mycousnine enamine. 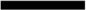 ^1^H-^1^H COSY 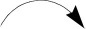 HMBC.

**Figure 3:**
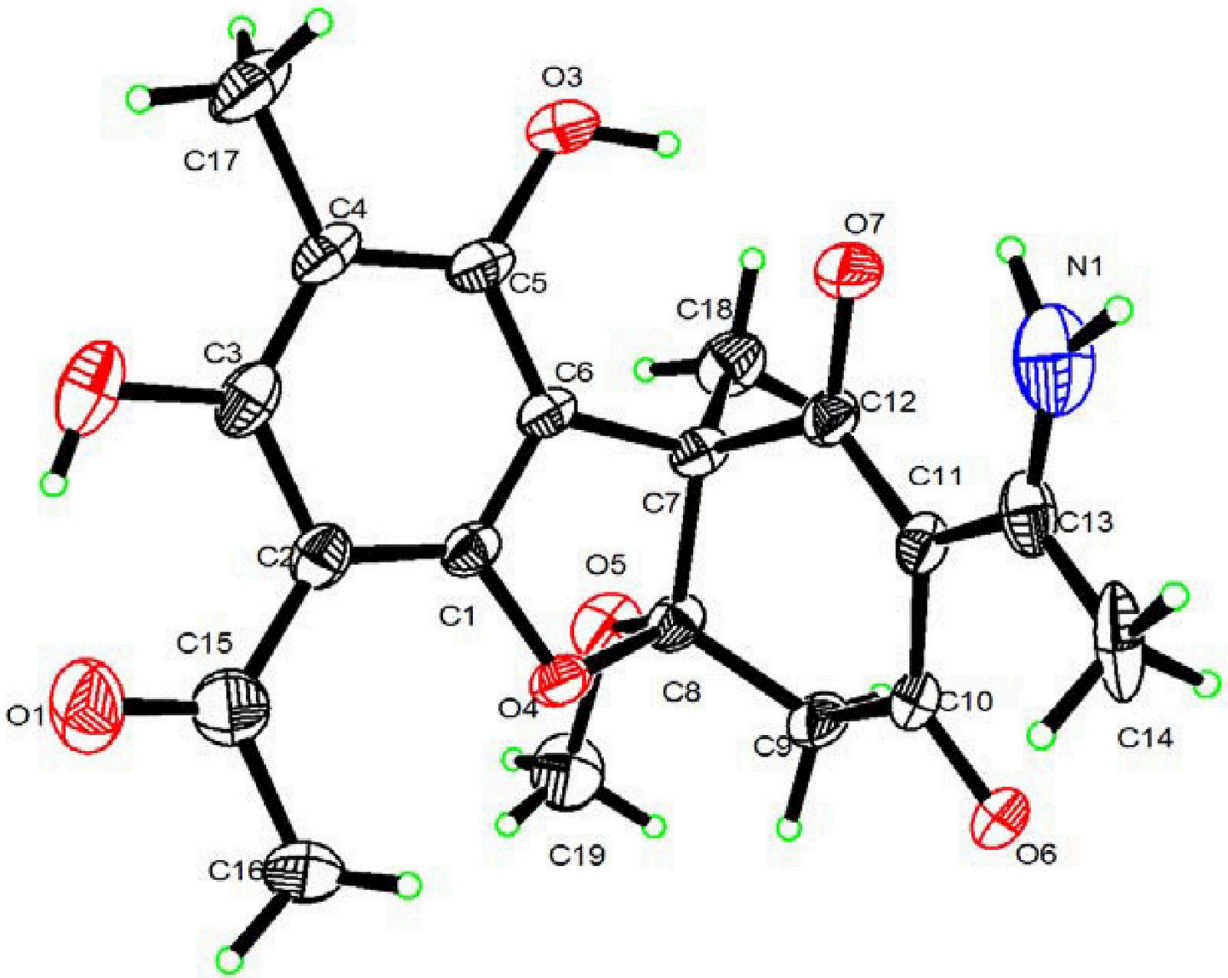
X-ray crystallographic structure of (-)mycousnine enamine.

**Figure 4:**
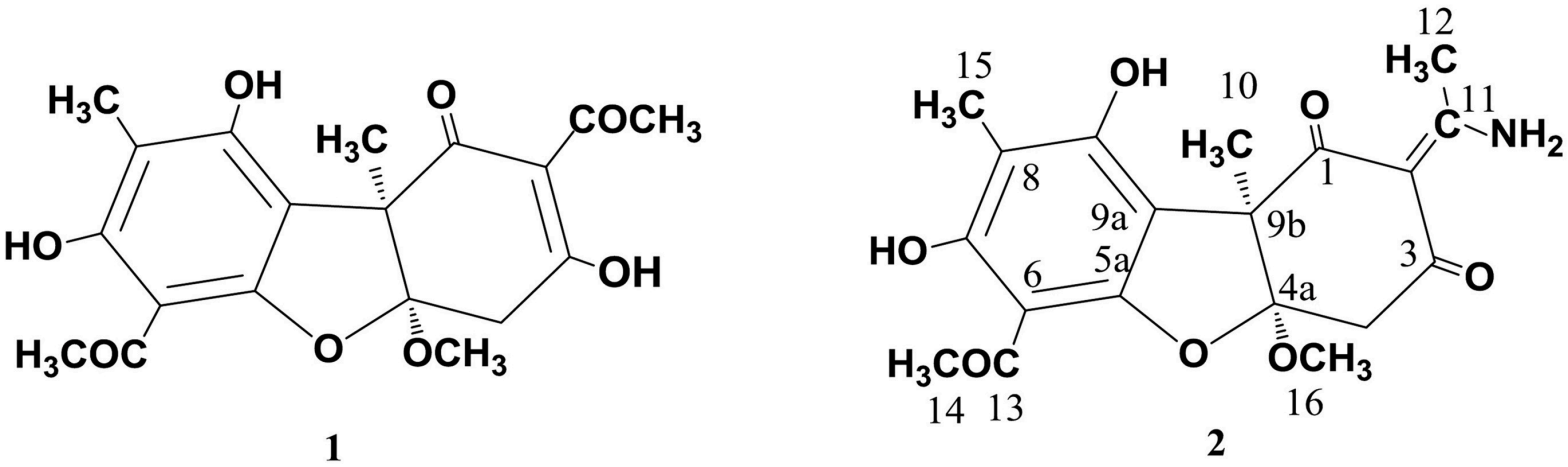
Structures of (-)mycousnine (**1**) and (-)mycousnine enamine (**2**).

As already reported above, and consistent with the literature (Sassa et al., 1989; Sassa and Igarashi, 1990) the major metabolite produced by *M. nawae* (compound **1**) was identified as (-)-mycousnine by spectroscopic and physical analysis (NMR data Refer to Table 1, Physical constants and HRESIMS spectroscopic data are given in Figure S6), (-) mycousnine is a dibenzofurane metabolite, and its β -diketone system is highly reactive and thus unstable (Kutney and Sanchez, 1976). In order to produce a stable heterocyclic derivate of (-)mycousnine, we generated an amine adduct by the reaction of the ketone with ammonium hydroxide. The most potent arising compound (Compound **2**) was screened assaying their bioactivity. It was isolated as yellow needles, and their ^1^H and ^13^C NMR spectra (Table 1) were very similar to those of (-) mycousnine except that additional enamine group signals at δ 6.91, 11.92 in ^1^H NMR were present. In addition, the key correlations of H-N/H-12 in ^1^H-^1^H COSY experiment and H-12/C-11 in HMBC spectra (Figure 2) supported the presence of a novel enamino diketo system in ring C of the (-)mycousnine amine adduct. The absolute configuration of (-)mycousnine amine was similar to that of (-)mycousnine (the optical rotations [α]20 D were −136°and −91° for (-)mycousnine amine and (-)mycousnine, respectively). Finally, major structural features of (-)mycousnine amine, including absolute configuration, were confirmed by X-ray diffraction of a crystal grown from methanol and water (4:1) (Figure 3), and the structure therefore determined to be (4a*R*,9b*S*,*E*)-6-acetyl-2-(1-amino-ethylidene)-7,9- dihydroxy-4a-methoxy-8,9bdimethyl-4,4a- dihydrodibenzo [b,d]furan-1,3(2H,9bH)-dione (Figure 4). Consequently, we named the isolated and characterized compound (-) mycousnine enamine. The physical constants, HRESIMS spectroscopic, crystallographic and NMR data are supplied as supplementary data and Figures S1–S6.

**Table 1:** ^1^H (500 MHz) and ^13^C NMR (125 MHz) data of (-)mycousnine (**1**), (-)mycousnine enamine (**2**) and HMBC correlations of **2** in CDCl_3_ (*J* in Hz, δ ppm).

**Position**	**(1)**		**(2)**		
	**δ_*C*_**	**δ_*H*_**	**δ_C_^a^**	**δH^a^**	**HMBC of (2)**
1	197.7		199.2		
2	110.7		106.7		
3	194.6		193.2		
4	37.9	3.17d	42.1	3.11	C-3; C-4a
		(18.0)		(18.0)	
		3.17d		3.11	C-3; C-4a
		(18.0)		(18.0)	
4a	109.6		110.3		
5a	156.6		156.6		
6	101.8		101.5		
7	163.4		162.9		
8	107.2		107.0		
9	159.1		159.6		
9a	105.3		105.6		
9b	59.8		60.3		
10	17.3	1.63 s	18.3	1.59s	C-1; C-4a; C-9a; C-9b
11	202.4		175.2		
12	27.4	2.56 s	25.6	2.52s	C-11
13	201.0		201.3		
14	31.2	2.59 s	31.2	2.60s	C-13
15	7.2	2.04 s	7.2	2.03s	C-7; C-8; C-9
16	51.3	3.54 s	51.4	3.56s	C-4a
N-H_1_				6.91brs	
N-H_2_				11.92brs	
7-OH				13.40brs	C-6; C-7; C-8; C-9
9-OH				10.56brs	C-9; C-9a

a *Signals were assigned by the DEPT, HSQC, HMBC, and ^1^H - ^1^H COSY spectra*.

### (-)mycousnine enamine selectively inhibits T cell proliferation

**Figure 5:**
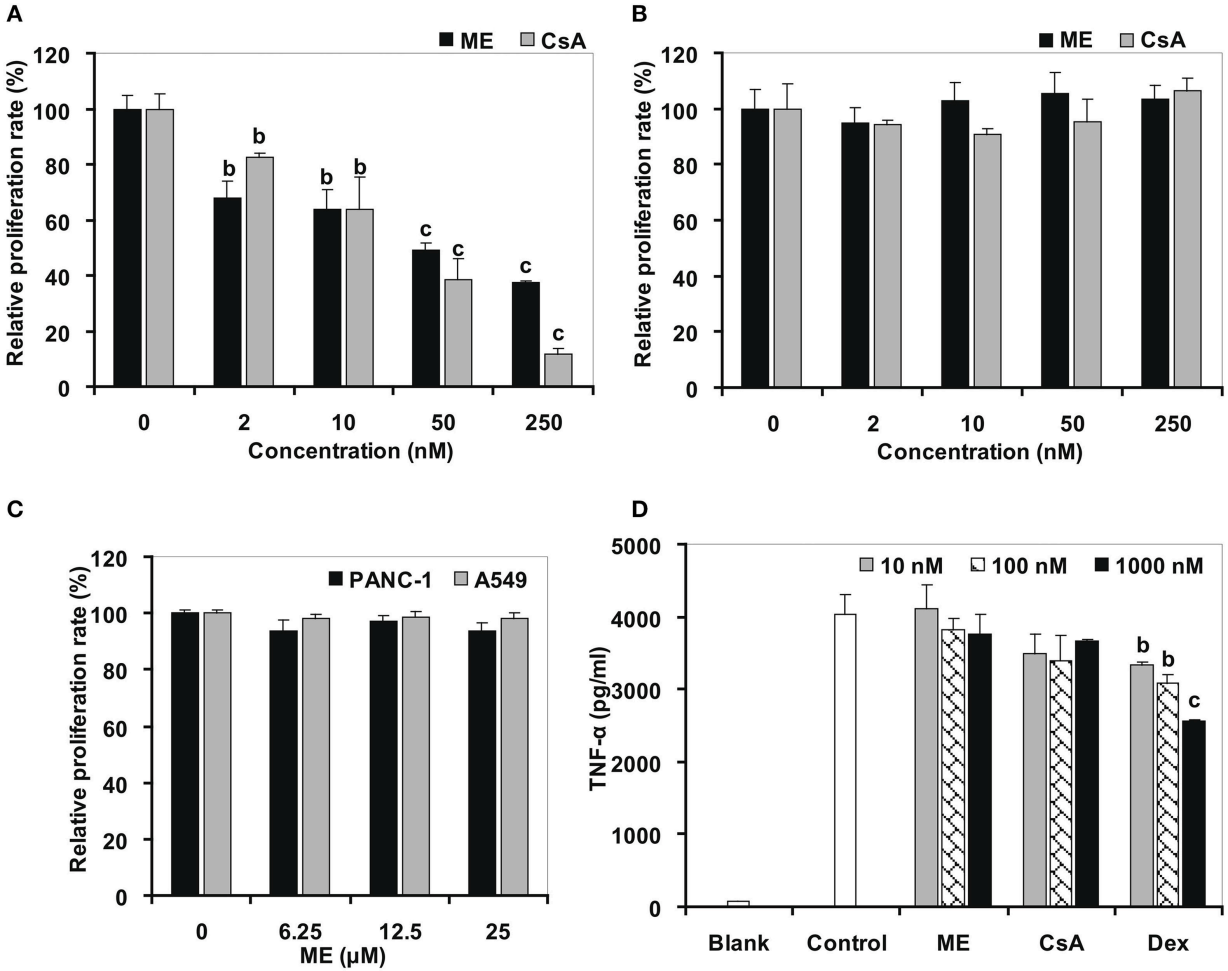
(-)mycousnine enamine (ME) selectively inhibited T cells proliferation. **(A)** Con A induced T cells proliferation. **(B)** LPS induced B cells proliferation. Splenocytes were incubated with (-)mycousnine enamine in the present of Con A or LPS (2 μg/ml) for 48 h, cell proliferation was determined using MTT assay. The values are presented as means ± S.D. (*n* = 4). Significant differences with Control group (0 nM) were designated as ^*b*^*P* < 0.01 and ^*c*^*P* < 0.001. **(C)** PANC-1 and A549 cells proliferation. Cells were incubated with (-)mycousnine enamine for 48 h, cell proliferation was tested by MTT method. The values are presented as means ± S.D. (*n* = 4). **(D)** LPS induced TNF-α production of Raw264.7 cells. Raw264.7 cells were incubated with (-)mycousnine enamine in the present of LPS (5 ng/mL) for 24 h, the cultured supernatants were gained for the detection of TNF-α by commercial ELISA kits. The values are presented as means ± S.D. (*n* = 3). Significant differences with Control group were designated as ^*b*^*P* < 0.01 and ^*c*^*P* < 0.001.

The plant lectin concanavalin A (Con A) is a mitogen that directly triggers the activation and proliferation of T cells by interacting with diverse receptors containing sugars, glycoproteins, or glycolipids (Li et al., 2010), and by stimulating the energy metabolism in T cells (Krauss et al., 1999). By using the 3-[4,5-dimethylthylthiazol-2-yl]-2,5-diphenyltetrazolium bromide (MTT) assay, we found that the original (-) mycousnine only slightly inhibited the stimulation of mouse T cells proliferation (at concentrations >10 μM; data no shown), whereas (-)mycousnine enamine significantly inhibited it in a concentration-dependent manner at the nM range (*P* < 0.01 or *P* < 0.001) (Figure 5A). On the other hand, (-)mycousnine enamine did not inhibit lipopolysaccharide (LPS)-induced B cell proliferation (Figure 5B). These data are comparable to the well-known calcineurin inhibitor cyclosporine A, which we tested as a control, and which significantly inhibited Con A-induced T cell proliferation at concentrations >2 nM (*P* < 0.01 or *P* < 0.001), but did not exhibit any effect on LPS-induced B cell proliferation (Figure 5B).

In order to further support that (-)mycousnine enamine is a specific inhibitor of T cell activation, we tested its effect on the proliferation of cancer cells and the activation of macrophage. As shown in Figure 5C, (-)mycousnine enamine had no effect on the proliferation of PANC-1 and A549 cancer cells at concentrations <25 μM. It also did not inhibit LPS-induced TNF-α production in RAW264.7 macrophages at 1 μM concentrations. Similar data were again obtained for cyclosporine A (CsA) (Figure 5D). However, dexamethasone (Dex), a non-selective anti-inflammatory drug, significantly inhibited TNF-α production in macrophage at the concentrations of 10–1,000 nM (*P* < 0.01 or *P* < 0.001), which suggested that the effects of CsA and (-)mycousnine enamine are similar, and the latter thus selectively inhibits T-cell activation *in vitro*.

**Figure 6:**
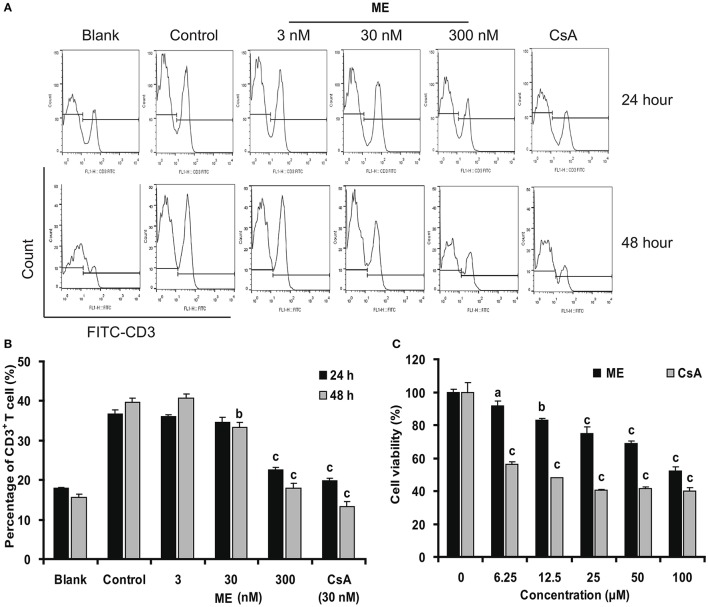
(-)mycousnine enamine inhibited CD3^+^ T cells proliferation. **(A)** Con A- stimulated splenocytes were incubated with (-)mycousnine enamine for 24 or 48 h. The cells were gathered and stained with FITC-anti-CD3. After washed with PBS, samples were detected by flow cytometry analysis. **(B)** The values are presented as means ± S.D. (*n* = 3). Significant differences with Control group were designated as ^*b*^*P* < 0.01 and ^*c*^*P* < 0.001. **(C)** Cytotoxicity of (-)mycousnine enamine on splenocytes. Splenocytes were incubated with (-)mycousnine enamine for 48 h, the cell viability was determined using MTT method. The values are presented as means ± S.D. (*n* = 4). Significant differences with Control group (0 μM) were designated as ^*a*^*P* < 0.05, ^*b*^*P* < 0.01, and ^*c*^*P* < 0.001.

The inhibition of (-)mycousnine enamine on T cell proliferation was further confirmed by flow cytometry analysis. As shown in Figures 6A,B, Con A significantly stimulates the percentage of CD3^+^ T cells in mice splenocytes. (-)mycousnine enamine, at concentrations between 30 and 300 nM, significantly inhibited this proliferation of CD3^+^ T cells (*P* < 0.01 or *P* < 0.001). To exclude the possibility that this would be due to a general cytotoxic effect of (-)mycousnine enamine, we also measured its effect on the viability of splenocytes viability using the MTT method. The results showed that (-)mycousnine enamine had no effect on cell viability up to 6.25 μM, but indeed decreased cell viability at higher concentrations (Figure 6C). Interestingly, this cytotoxicity is lower than that of cyclosporine A. and therapeutic index (TC50/ EC50) of (-)mycousinic enamine (4,463.5) is much higher than that of cyclosporine A (885.0). These results indicate that the observed selective suppression of T cell proliferation by (-)mycousnine enamine is not due to its cytotoxicity.

### (-)mycousnine enamine inhibits CD25 and CD69 surface expressions in activated T cells

**Figure 7:**
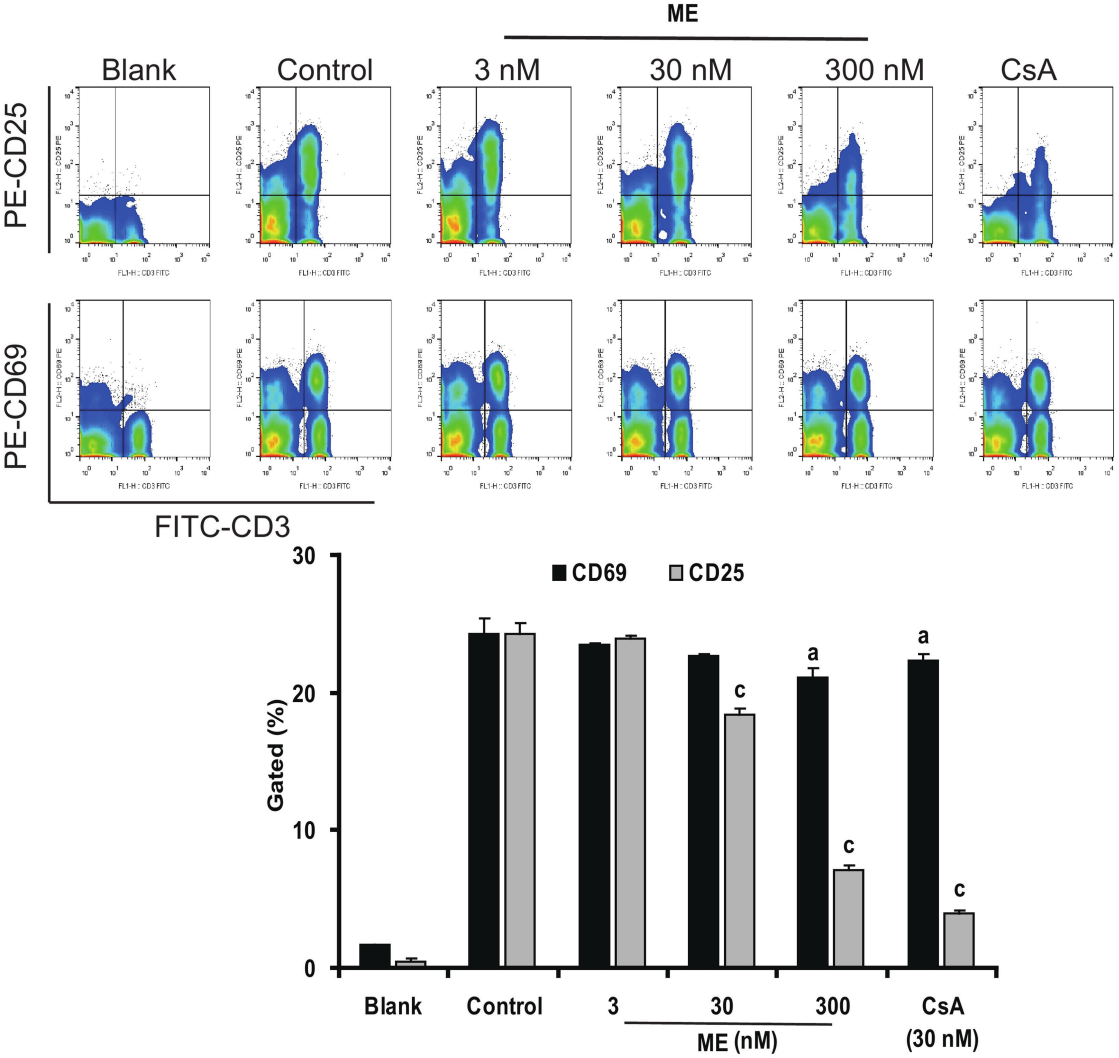
Inhibition of (-)mycousnine enamine on T cells surface molecules CD69 and CD25 expressions. Con A- stimulated splenocytes were incubated with (-)mycousnine enamine for 6 h and 24 h, the cells were collected and stained with FITC-anti-CD3 plus PE-anti-CD69 and FITC-anti-CD3 plus PE-anti-CD25, respectively. After washed with PBS, samples were analyzed through flow cytometry analysis. The values are presented as means ± S.D. (*n* = 3). Significant differences with Control group were designated as ^*a*^*P* < 0.05, and ^*c*^*P* < 0.001.

CD25 and CD69 are usually considered as the activation marker of T-cells (Wieland and Shipkova, 2016). To have a closer look at the mechanism of (-)mycousinic enamine on T cells activation, the surface expression levels of CD69 and CD25 were determined by flow cytometry analysis. As shown in Figure 7, CD69 and CD25 were up-regulated in T cells incubated with Con A for 6 h and 24 h, respectively. (-)mycousnine enamine, at concentrations of 30 and 300 nM, significantly inhibited the expressions of CD69 and CD25 in Con A-stimulated T cells (*P* < 0.05 or *P* < 0.001). These data suggest that (-)mycousnine enamine can inhibit the activation of T cells *in vitro*.

### (-)mycousnine enamine suppresses cytokine expression and production in activated T cells

**Figure 8:**
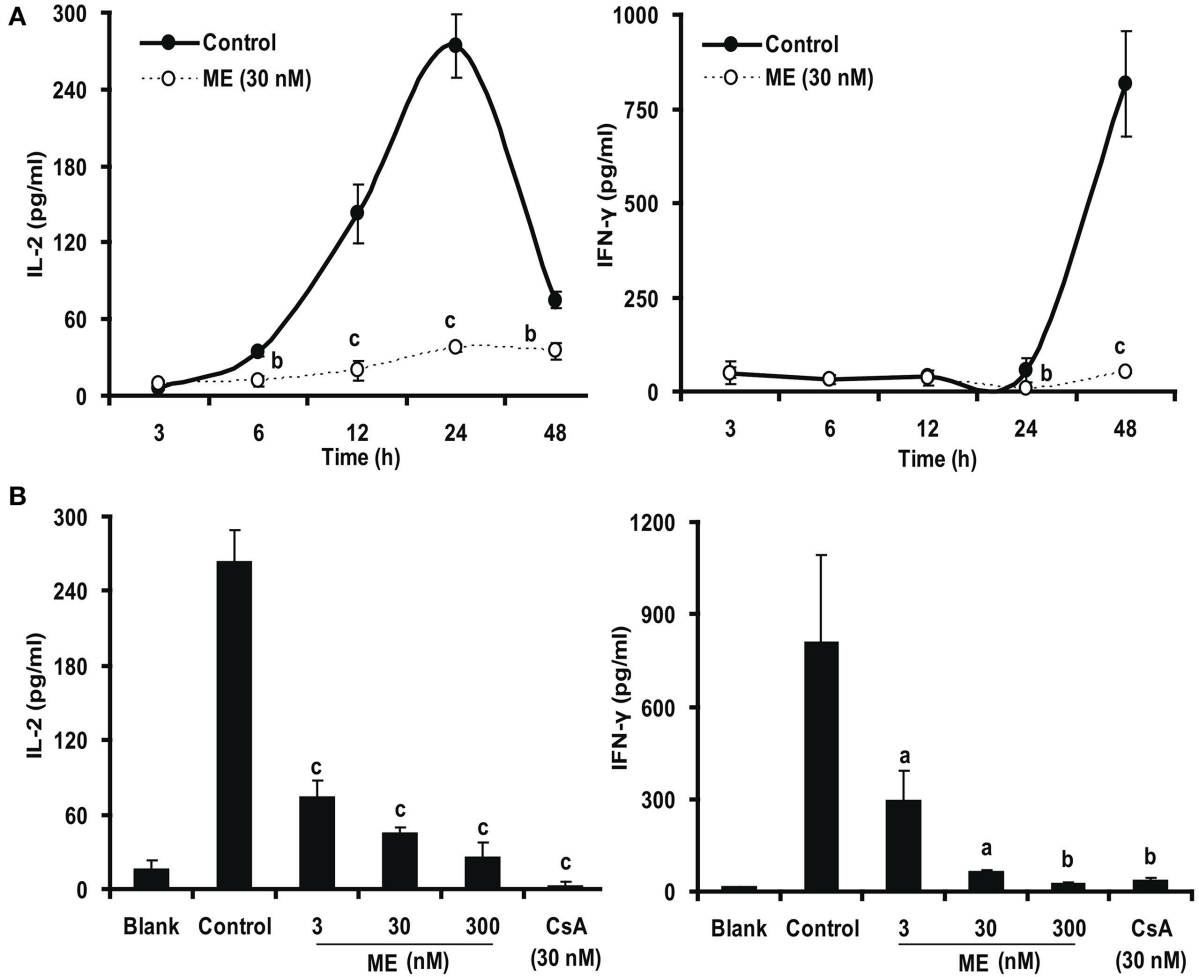
(-)mycousnine enamine inhibited cytokines IL-2 and IFN-γ production. **(A)** Time course effect of (-)mycousnine enamine on cytokines production. Con A-stimulated splenocytes were incubated with (-)mycousnine enamine for designated length of time, the cultured supernatants was collected for the detection of cytokines contents. **(B)** The dose-related effect of (-)mycousnine enamine on cytokines production. Con A-stimulated splenocytes were incubated with (-)mycousnine enamine for 24 or 48 h, the cultured supernatants was collected for the detection of IL-2 and IFN-γ respectively by commercial ELISA kits. The values are presented as means ± S.D. (*n* = 3). Significant differences with Control group were designated as ^*a*^*P* < 0.05, ^*b*^*P* < 0.01, and ^*c*^*P* < 0.001.

IL-2 and IFN-γ are the two main cytokines produced by T cells after activation (Millán and Brunet, 2016). We were thus interested whether (-)mycousnine enamine would also have an effect at their expression and formation. Therefore, we first quantified the concentrations of these two cytokines in the culture supernatant by ELISA. To this end, we first determined the kinetics of cytokine production from Con A-stimulated splenocytes in the absence or presence of (-)mycousnine enamine (30 nM). As shown in Figure 8A, the levels of IFN-γ increased in a time-dependent manner within 48 h, whereas the levels of IL-2 were increased and reached a peak level at about 24 h, and then declined. (-)Mycousnine enamine significantly suppressed the secretion of IL-2 and IFN-γ from the Con A- stimulated splenocytes within this incubation time (*P* < 0.05 or *P* < 0.001). Having established the optimal time point, we further investigated the dose-related effect of (-)mycousnine enamine on the secretion of IL-2 and IFN-γ from the Con A -stimulated splenocytes. The results, shown in Figure 8B, documented that the formation of cytokines IL-2 and IFN-γ by Con A-stimulated splenocytes was significantly reduced by (-)mycousnine enamine in concentration-dependent manner, ranging from 3 to 300 nM (*P* < 0.05, *P* < 0.01 or *P* < 0.001).

**Figure 9:**
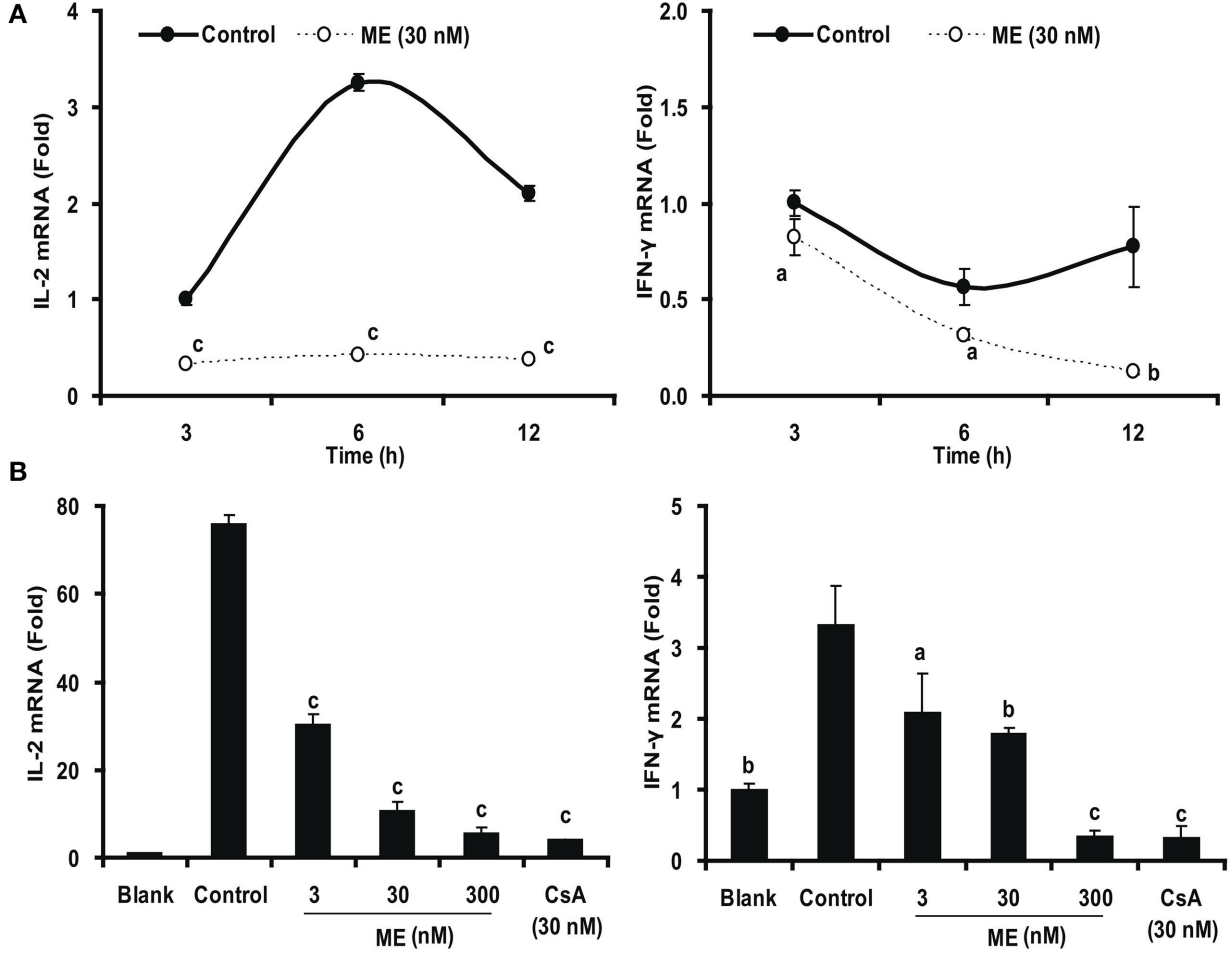
(-)mycousnine enamine inhibited mRNA expression of cytokines IL-2 and IFN4γ. **(A)** Time course effect of (-)mycousnine enamine on cytokines mRNA expression. Con A- stimulated splenocytes were incubated with (-)mycousnine enamine for designated length of time, the cells was collected for the detection of cytokines mRNA expression. **(B)** Dose-related effect of (-)mycousnine enamine on cytokines mRNA expression. Con A- stimulated splenocytes were incubated with (-)mycousnine enamine for 6 h, the cells was collected for the detection of IL-2 and IFN-γ mRNA expression by Real-time PCR. The values are presented as means ± S.D. (*n* = 3). Significant differences with Control group were designated as ^*a*^*P* < 0.05, ^*b*^*P* < 0.01, and ^*c*^*P* < 0.001.

In order to test whether this effect is due to an inhibition of cytokine gene expression, we determined their mRNA (IL-2 and IFN-γ) formation by real-time PCR. As shown in Figure 9, Con A stimulation of the splenocytes strongly up-regulated the expression of IL-2 and IFN-γ. This upregulation was significantly down-regulated by 30 nM (-)mycousnine enamine within the specific 3-12 h of incubation (*P* < 0.05, *P* < 0.01 or *P* < 0.001). Consistent data were obtained using different (-)mycousnine enamine concentrations (3–300 nM; *P* < 0.05, *P* < 0.01 or *P* < 0.001).

## Discussion

Because of the unique interaction between the endophytic fungi and their host plants, several efforts have been made to identify bioactive substances produce by these organisms (Kaul et al., 2012; Yuan et al., 2014; Lai et al., 2016). Indeed, endophytic fungi in medicinal plants are potential sources of novel bioactive secondary metabolites, and could contribute to the pharmacological effects of these plants. Evidence for this has been established with the discovery of taxol, an antitumor product synthesized by fungal endophyte in *Taxus brevifolia* (Strobel et al., 1996). In recent decades, several novel metabolites have been identified from endophytic fungi in medicinal plant with immunosuppressive activity (Kumar et al., 2005; Sun et al., 2012; Song et al., 2013). Previous studies have demonstrated that *Smilax china* L. is of medical importance and dozens of novel products have been isolated from it (Fan et al., 2014; Zhao et al., 2016). However, little attention has been paid to explore its endophytic fungi and the metabolites produced by them. Here, we have for the first time isolated an endophytic fungus—*M. nawae* ZJLQ129—in *Smilax china* L. and characterized its main metabolite.

So far, several bioactive compounds from fungal strains of the genus *Mycosphaerella* have been reported, including the antibiotics asteromine (Arnone et al., 1995) and hydroxytetradecatrienoic acid (Arnone et al, 1998), the phytotoxins rubellin C (Arnone and Nasini, 1989) and 5-hydroxymethylfuran-3-carboxylic acid (Assante et al., 2009), cercosporin which exhibits antitumor activity (Morgan et al., 2009). Sassa et al. (1989) isolated (-)mycousnine from a strain of *M. nawae* that caused circular leaf spot disease in Japanese persimmon *Diospyros ka*. They also showed that (-)mycousnine exhibited antiviral activity but in the μM concentration range (Sassa and Igarashi, 1990). Here we eventually isolated the same species as an endophyte of *Smilax china* L, and found that it produces the same metabolite, (-)mycousnine.

Since the first isolation of usnic acid (UA)—a highly functionalized dibenzofuran from lichens (Stark et al., 1950), numerous dibenzofuran metabolites have been isolated from a variety of lichens and endophytic fungi (Sugawara et al., 1991; Hoffman et al., 2008; Furukawa et al., 2010; Millot et al., 2016). Like UA, these metabolites are today well known to possess various pharmacological activities such as antineoplastic (Takai et al., 1979), antibacterial (Ingolfsdottir et al., 1998), anti-inflammatory (Vijayakumar et al., 2000), selective inhibition of protein kinase C1 (Sussman et al., 2000; Shih and Ye, 2004), antifungal activity (Hoffman et al., 2008) and the ability to lower the plasma glucose concentration (Furukawa et al., 2010). Because of the wide array of biological activity displayed by these dibenzofuranes, there has been continuing interest in UA derivatives. To this end, we prepared (-)mycousnine enamine—a dibenzofuran compound—semisynthetically and found that it exhibited a stronger selective inhibition of T cells proliferation than the original isolated nature product (-)mycousnine after structural modification. Compare these two compounds, the only difference is introduction of the enamino diketo of synthetic (-)mycousnine enamine. Our data therefore show that this group was vital important for its immunosuppressive effect. Since little has been done to the immunosuppressive activity of these dibenzofuran compounds, further structure-activity relationship investigation was extremely essential in future work.

T cells are a potential and preferred target for treatment of autoimmune diseases and inflammatory disorders (Azizi et al., 2016). Their activation involves the induction of gene transcription, the expression of new cell surface molecules, the secretion of cytokines, and the induction of mitotic activity resulting in colonel proliferation. Calcineurin inhibitors Cyclosporin A and Tacrolimus inhibit the phosphatase activity of calcineurin and thereby suppress the translocation of the nuclear factor of activated T cells, NFAT, and thus block T-cell activation (Krenzien et al., 2017). Calcineurin inhibitors are the cornerstones of modern immunosuppression research. Since the launching of CsA in 1984, calcineurin inhibitors-based therapies are the main immunosuppressive protocol for almost all types of organ transplantations (Cohen et al., 1984; Macleod and Thomson, 1991). These drugs also greatly improved the perspectives for other autoimmune and inflammatory disorders (Wada et al., 2015). However, because they are only therapeutically applicable in a narrow concentration window, their uses are associated with many side-effects, including nephrotoxicity, hypertension, neurological disorders, new-onset diabetes, *de novo* cancers, and dyslipidemia (Malvezzi and Rostaing, 2015). Consequently, many efforts have been done to develop less toxic and more effective immunosuppressants (Webber and Vincenti, 2016), but until now, no small molecules that could substitute the calcineurin inhibitors have been found.

In the present study, we identified (-)mycousnine enamine, a novel dibenzofurane derivative of (-)mycousnine from the endophytic fungus *M. nawae* to possess immunosuppressive activity by specifically interfering with the T cell response. Similar to cyclosporine A, (-)mycousnine enamine selectively inhibited T cell proliferation, suppressed the expression of surface molecules (CD25 and CD69) and cytokines (IL-2 and IFN-γ) in activated T cells, and it did not show any effect on the proliferation of B cell and cancer cells (PANC-1 and A549) or the activation of macrophage. However, the cytotoxicity of (-)mycousnine enamine was lower than that of CsA, and therapeutic index (TC_50_/EC_50_) of (-)mycousinic enamine (4,463.5) is much higher than that of cyclosporine A (885.0).

In conclusion, the present study demonstrated that (-)mycousnine enamine, derived from (-)mycousnine in the fungal endophyte *M. nawae* is a novel immunosuppressant with high effectivity, low toxicity and high selectivity. These finding justify further studies on its activity *in vivo* as well as its mechanism of action.

## Author contributions

LW, CZ, FC and FL were responsible for the idea and concept of the paper. JW, JC, JJC, JS, and XF collected and analyzed the data, LW, CZ, and FC wrote the manuscript. CK, LW and FC revised the manuscript.

### Conflict of interest statement

The authors declare that the research was conducted in the absence of any commercial or financial relationships that could be construed as a potential conflict of interest. The reviewer BF and handling Editor declared their shared affiliation, and the handling Editor states that the process nevertheless met the standards of a fair and objective review.

## Abbreviations

BI, Bayesian inference, Con A: concanavalin A
CsA: cyclosporine A
Dex: dexamethasone
LPS: lipopolysaccharide
ME: (-)mycousnine enamine
MTT: 3-[4,5-dimethylthylthiazol-2-yl]-2,5- diphenyl-tetrazolium bromide
PDA: potato dextrose agar
WA: water agar.
