# Supplementary material for: A Novel Derivative of (-)mycousnine Produced by the Endophytic Fungus Mycosphaerella nawae, Exhibits High and Selective Immunosuppressive Activity on T Cells
Source: Front Microbiol. 2017 Jul 5;8:1251. doi: 10.3389/fmicb.2017.01251 (PMC5496962; doi:10.3389/fmicb.2017.01251)
Supplement: Supplementary file 1 [file Presentation1.PDF]

Supplementary Material

Article Title: A novel derivative of (-)mycousnine produced by the endophytic fungus *Mycosphaerella nawae,* exhibits selective immunosuppressive activity on T cells

Li-Wei Wang[1], Jin-Liang Wang[2], Jing Chen[2], Jia-Jie Chen[2], Jia-Wei Shen[1], Xiao-Xiao Feng[2], Zhen-zhu Su[2], Christian P. Kubicek[4], Fu-Cheng Lin[2], Chu-Long Zhang[2]*and Feng-Yang Chen[3]*

*Correspondence:

Li-Wei Wang, Jia-Wei Shen, Department of Pharmaceutical Science, College of Medical Science, Hangzhou Normal University, Hangzhou 310036, China

Jin-Liang Wang, Jing Chen, Jia-Jie Chen, Xiao-Xiao Feng, Fu-Cheng Lin, Chu-Long Zhang, State Key Laboratory of Rice Biology, Institute of Biotechnology, Zhejiang University, Hangzhou 310058, China

Feng-Yang Chen, Zhejiang Academy of Medical Sciences, Hangzhou 310007, China

Christian P. Kubicek, Institute of Chemical Engineering, Vienna University of Technology, 1060 Vienna, Austria

The e-mail address, telephone and fax numbers of the corresponding author:

e-mail: clzhang@zju.edu.cn, Tel.: +86 571 88982291; Fax: +86 571 88982183.

1. SUPPLEMENTARY DATA

1.1 Physical constants, HRESIMS spectroscopic data of (-)mycousnine (1) and (-)mycousnine enamine (2)

(-)mycousunine (**1**), yellow needle, mp 86-89 ºC (MeOH), $[\alpha]_D^{20}$= -91°(C 0.18, $CHCl_3$); HRESIMS m/z: 376.1158 M+ (calcd for $C_{19}H_{20}O_8$ 376.1158).

(-)mycousnine enamine (**2**), yellow needle, mp 206-207 ºC (Acetone), yellow needle, mp 206-207 ºC (Acetone), $[\alpha]_D^{20}$ -136°(c = 0.1, MeCN), HRESIMS m/z: 376.1399 $[M+H]^+$ (calcd for $C_{19}H_{21}O_7N$ 376.1399).

1.2 Crystal data of (-)mycousnine enamine (2)

$C_{19}H_{21}NO_7$, M_r = 375.37; space group P212121, a = 10.9230 (5) Å, b = 11.1739 (5) Å, c = 15.0047 (7) Å, V = 1831.36 (15), $Å^3$, Z =4, D_x = 1.361 $mg.m^{-3}$. μ(Cu K)= 0.88 mm^{-1}, F(000) = 784. Crystal dimensions: (0.15 × 0.10 × 0.10 mm), independent reflections:2981(R_{int} = 0.047). The final R1 values were 0.054, $wR(F^2)$ = 0.128, Flack parameter: -0.2 (4). Crystallographic data for (-)mycousnine enamine (2) in this paper have been deposited with the Cambridge Crystallographic Data Centre as supplementary publication number CCDC 1512383. Copies of the data can be obtained free Cambridge Crystallographic Data Centre at www.ccdc.cam.ac.uk/data_request/cif.

2. SUPPLEMENTARY FIGURE

2.1 Figure S1. ^1H NMR spectrum of (-)mycousnine enamine (2)
2.2 Figure S2. ^{13}C NMR spectrum of (-)mycousnine enamine (2)
2.3 Figure S3. DEPT(135°) spectrum of (-)mycousnine enamine (2)
2.4 Figure S4. HSQC spectrum of (-)mycousnine enamine (2)
2.5 Figure S5. HMBC spectrum of (-)mycousnine enamine (2)
2.6 Figure S6. ^1H-^1H COSY spectrum of (-)mycousnine enamine (2)
