# Supplementary figures and images for: A Novel Derivative of (-)mycousnine Produced by the Endophytic Fungus Mycosphaerella nawae, Exhibits High and Selective Immunosuppressive Activity on T Cells
Source: Front Microbiol. 2017 Jul 5;8:1251. doi: 10.3389/fmicb.2017.01251 (PMC5496962; doi:10.3389/fmicb.2017.01251)

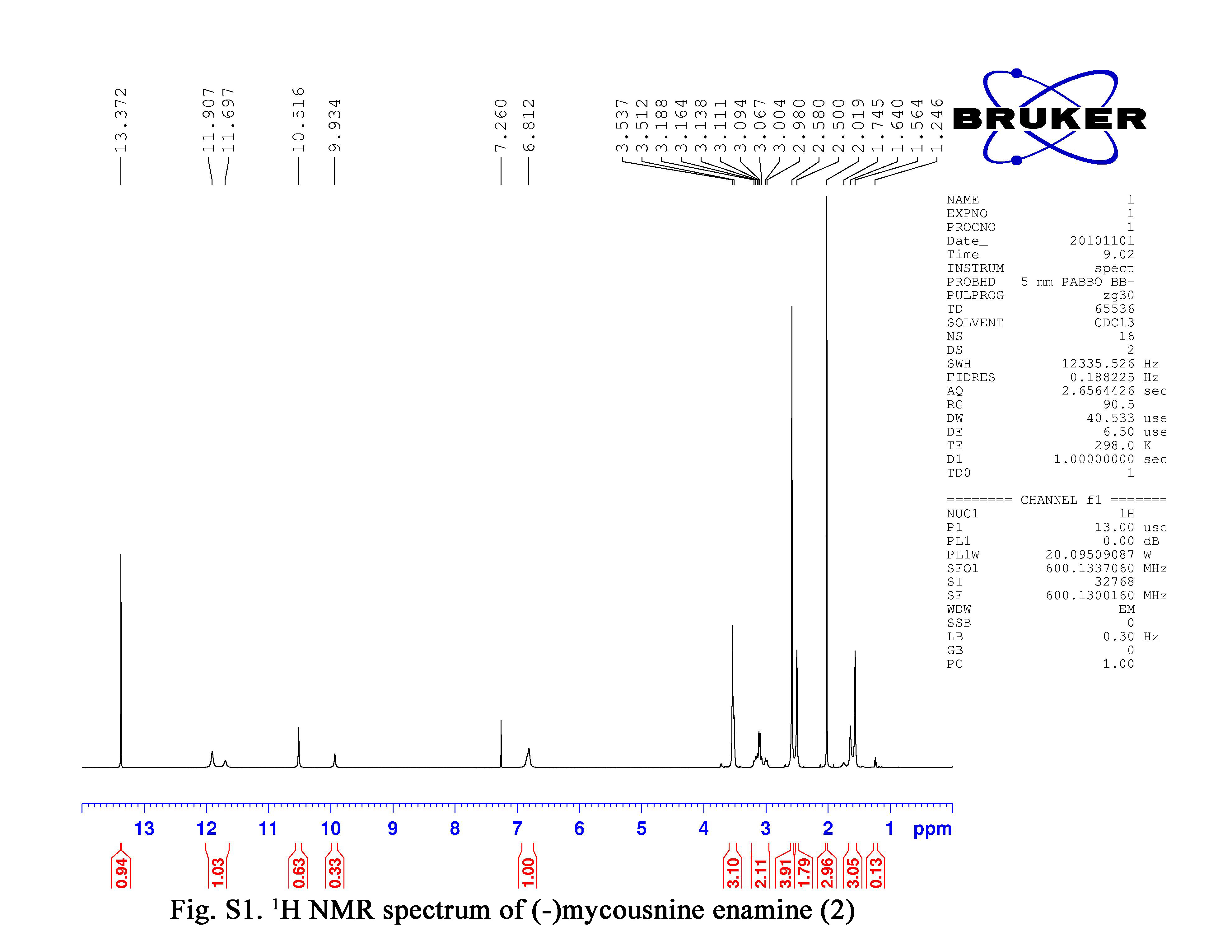

Supplement: Supplementary file 2 [file Image1.JPEG]

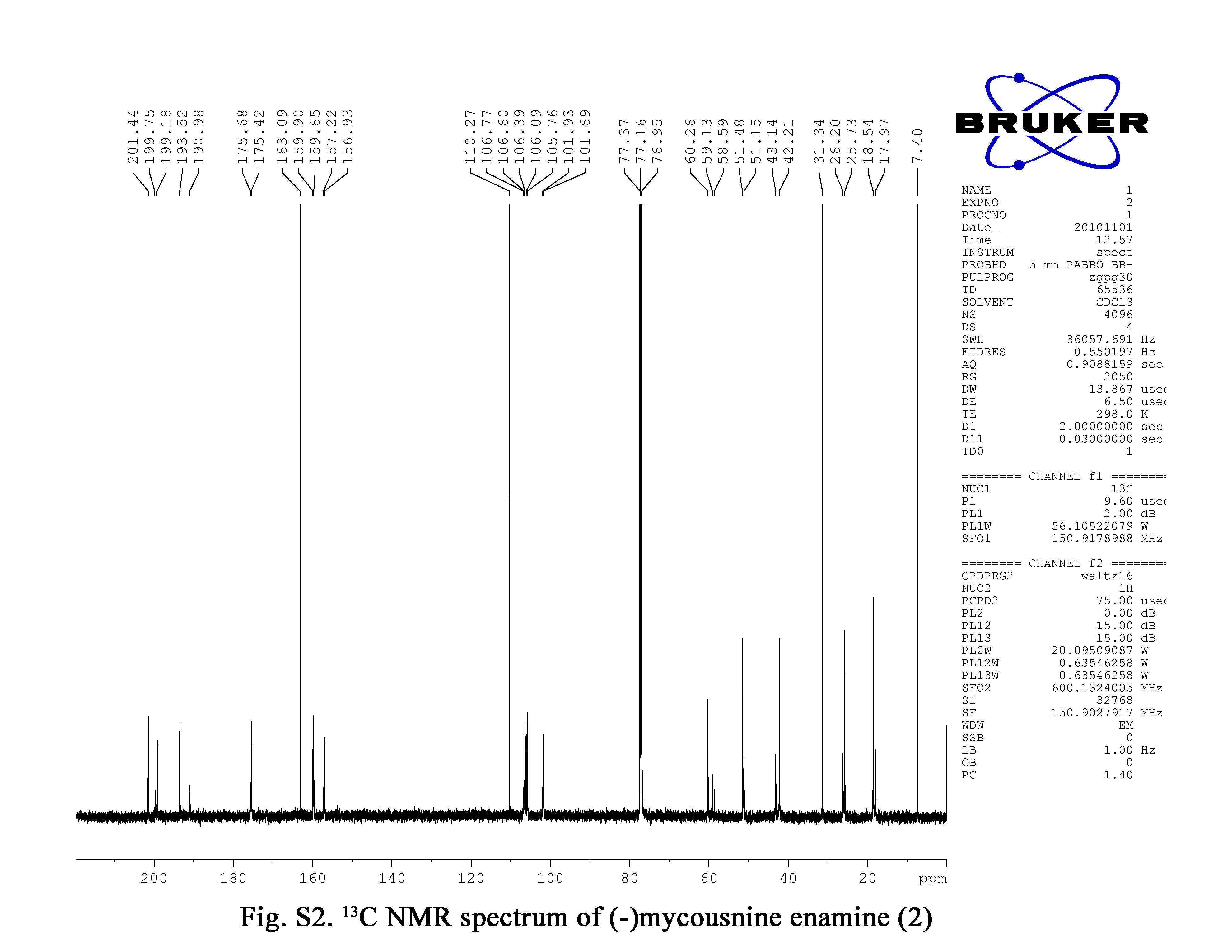

Supplement: Supplementary file 3 [file Image2.JPEG]

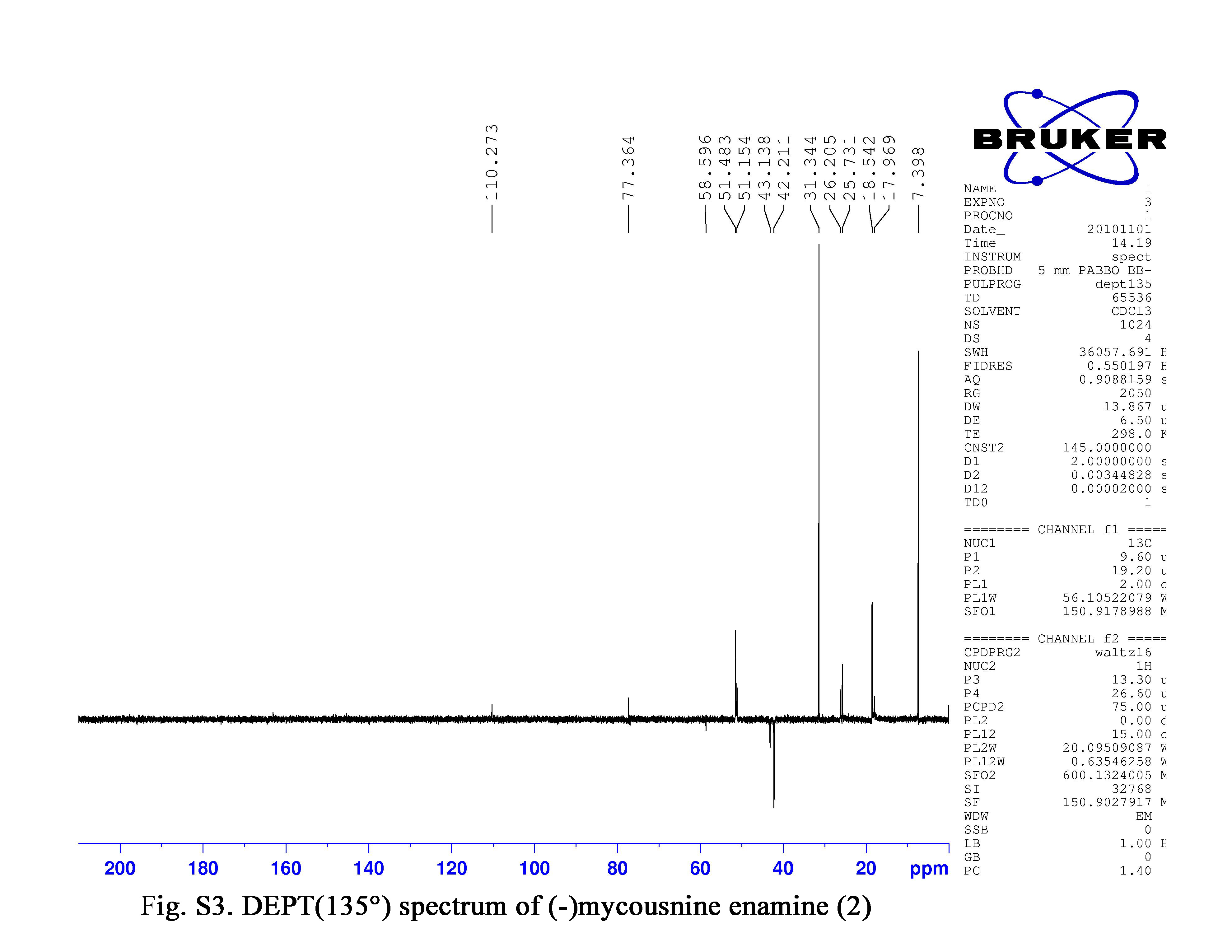

Supplement: Supplementary file 4 [file Image3.JPEG]

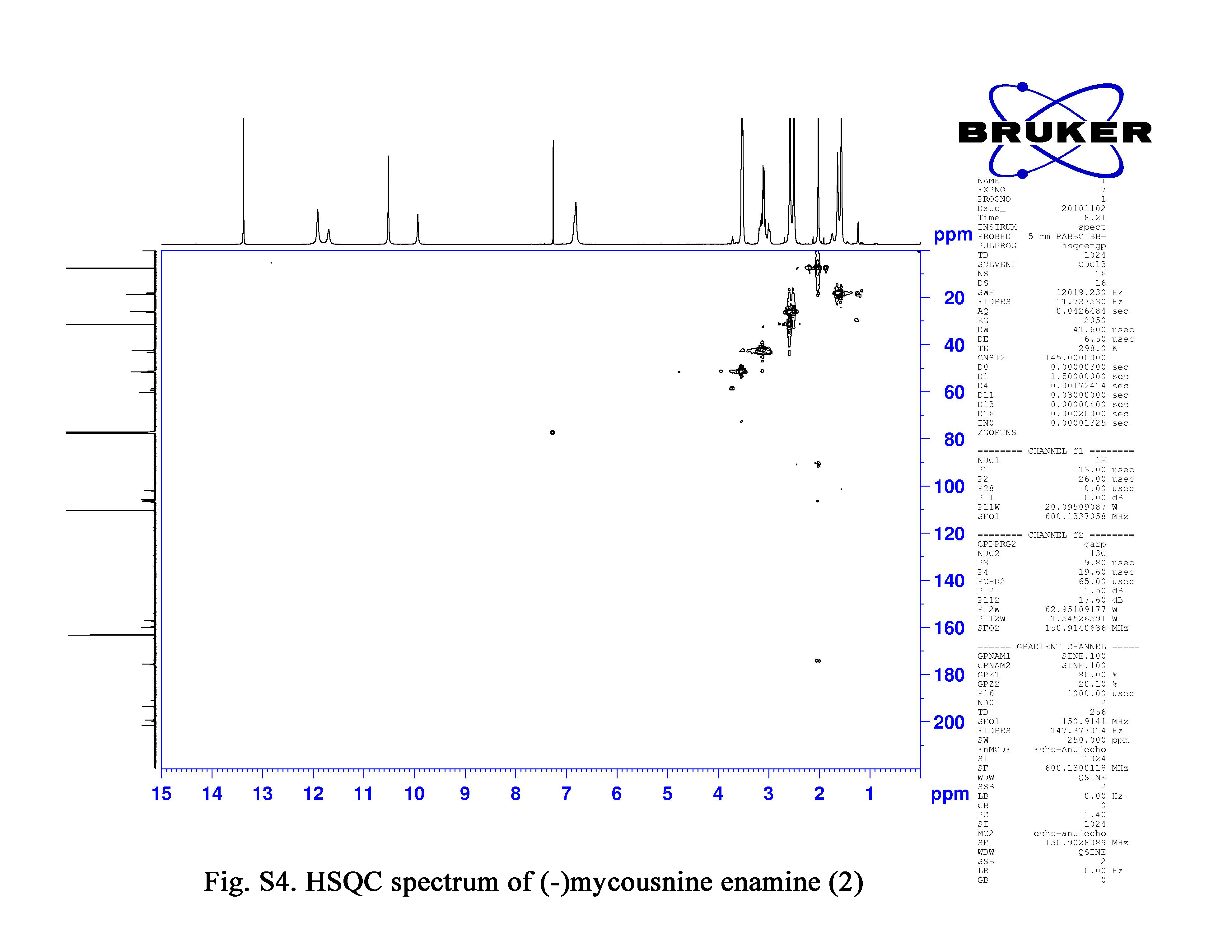

Supplement: Supplementary file 5 [file Image4.JPEG]

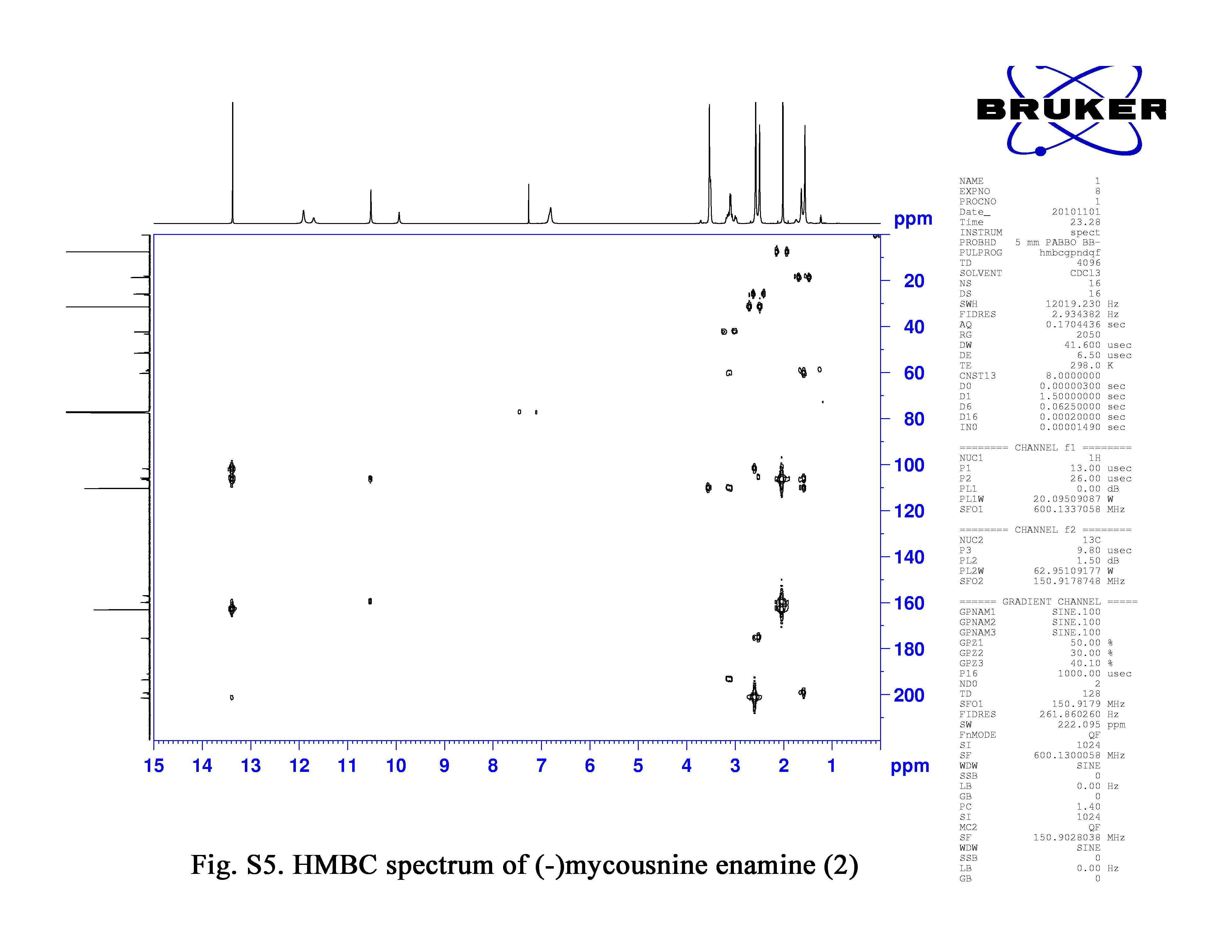

Supplement: Supplementary file 6 [file Image5.JPEG]

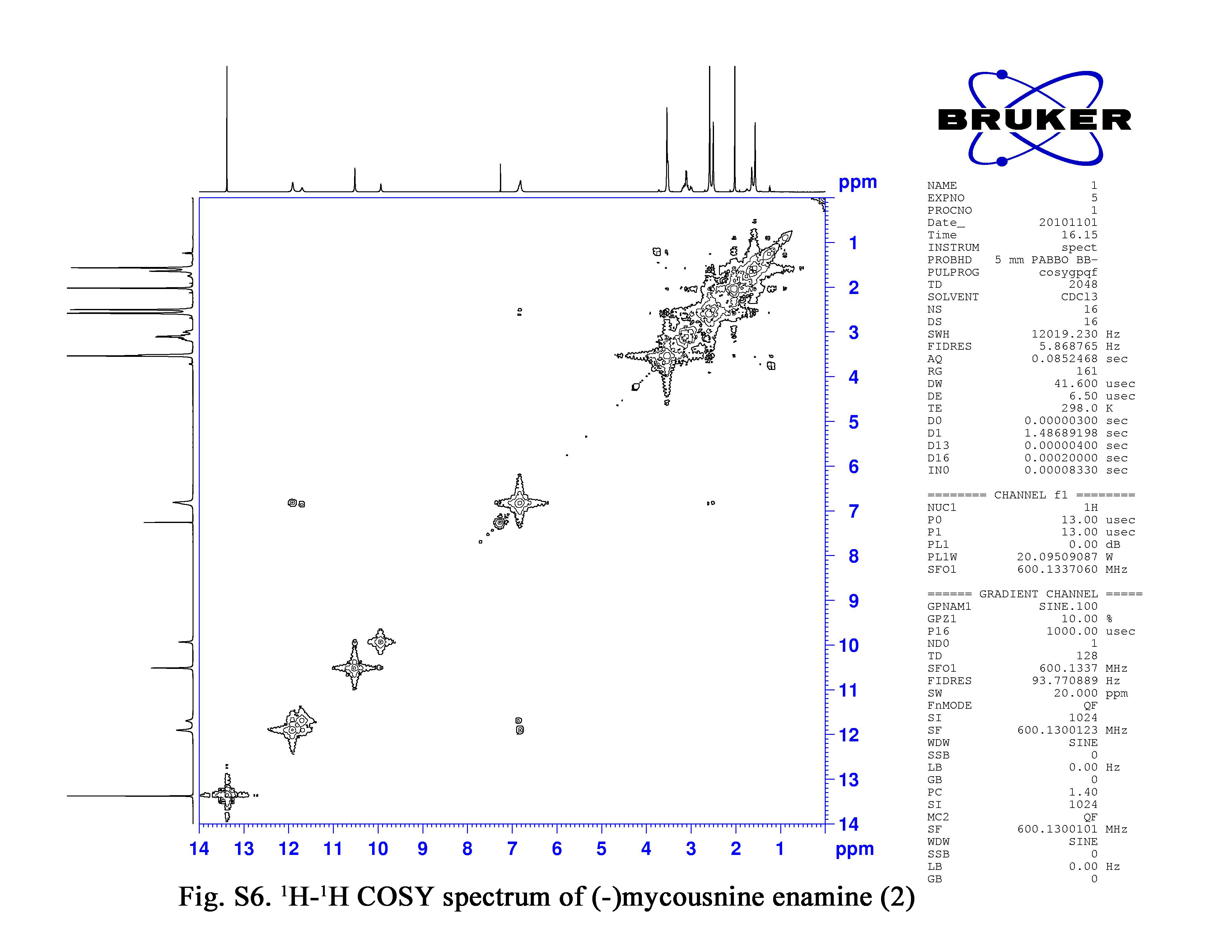

Supplement: Supplementary file 7 [file Image6.JPEG]
